# Epidemiology of Kaposi's sarcoma in Scotland, 1976–1996

**DOI:** 10.1038/sj.bjc.6690309

**Published:** 1999-04

**Authors:** D H Brewster, V Harris, R J Black, D J Goldberg

**Affiliations:** Scottish Cancer Intelligence Unit, Information & Statistics Division, Trinity Park House, Edinburgh, Scotland, EH5 3SQ, UK; Scottish Centre for Infection and Environmental Health, Clifton House, Clifton Place, Glasgow, Scotland, G3 7LN, UK

**Keywords:** AIDS, epidemiology, incidence, Kaposi's sarcoma, Scotland

## Abstract

Based on data from the Scottish Cancer Registry, the epidemiology of Kaposi's sarcoma (KS) in Scotland during the period 1976–96 is described. In males, the annual age-standardized incidence rate (World standard population) increased from less than 0.09 per 100 000 before 1986 to 0.44 in 1991 and then decreased to around 0.17. Peak incidence is now at ages 30–39 compared with ages 80+ during the period 1976–82; and by 1986–96 the standardized incidence ratio for the Health Board which includes Edinburgh had risen to almost four times the national level. These changes are largely consistent with the pattern of HIV infection in Scotland. However, in both sexes, relative to other neoplasms, and in international terms, KS remains rare in Scotland. For patients diagnosed during 1976–92, cumulative observed survival at 5 years was 8.7% at ages 0–49 compared with 49.8% at ages 50–84, reflecting the more aggressive course of AIDS-related KS, as well as the co-morbidity and competing causes of death associated with AIDS. © 1999 Cancer Research Campaign


*British Journal of Cancer* (1999) **79**(11/12), 1938–1942
© 1999 Cancer Research Campaign
Article no. bjoc.1998.0309

### Epidemiology of Kaposi's sarcoma in Scotland, 1976–1996

**DH Brewster[1], V Harris[1], RJ Black[1] and DJ Goldberg[2]**

[1]Scottish Cancer Intelligence Unit, Information & Statistics Division, Trinity Park House, Edinburgh EH5 3SQ, Scotland, UK; and [2]Scottish Centre for Infection and Environmental Health, Clifton House, Clifton Place, Glasgow G3 7LN, Scotland, UK

**Summary** Based on data from the Scottish Cancer Registry, the epidemiology of Kaposi's sarcoma (KS) in Scotland during the period 1976–96 is described. In males, the annual age-standardized incidence rate (World standard population) increased from less than 0.09 per 100 000 before 1986 to 0.44 in 1991 and then decreased to around 0.17. Peak incidence is now at ages 30–39 compared with ages 80+ during the period 1976–82; and by 1986–96 the standardized incidence ratio for the Health Board which includes Edinburgh had risen to almost four times the national level. These changes are largely consistent with the pattern of HIV infection in Scotland. However, in both sexes, relative to other neoplasms, and in international terms, KS remains rare in Scotland. For patients diagnosed during 1976–92, cumulative observed survival at 5 years was 8.7% at ages 0–49 compared with 49.8% at ages 50–84, reflecting the more aggressive course of AIDS-related KS, as well as the co-morbidity and competing causes of death associated with AIDS.

**Keywords:** AIDS; epidemiology; incidence; Kaposi's sarcoma; Scotland

Historically, Kaposi's sarcoma (KS) has been rare in the United Kingdom, both in relation to other neoplasms, and in international terms (Grulich et al, 1992). However, its profile has become more prominent since it was first reported among homosexual men in the United States (Centers for Disease Control, 1981) at the beginning of the global AIDS epidemic.

The spread of HIV and AIDS in Scotland has been documented in detail elsewhere (Goldberg et al, 1996, 1998). In summary, HIV first entered populations of homosexual/bisexual males and injecting drug users not later than 1982 and 1983, respectively (Goldberg et al, 1996). The majority of homosexual/bisexual males have been over 30 years old at the time of diagnosis and their progression to AIDS is likely to have been more rapid than the generally younger cohort of injecting drug users. Epidemic spread of HIV among injecting drug users occurred during 1983–86, particularly in Edinburgh and Dundee (Goldberg et al, 1996). By the end of 1997, a cumulative total of 2725 persons in Scotland had been identified as HIV antibody-positive (Goldberg et al, 1998). Just under half of cases were in injecting drug users, one third in homosexual/bisexual males, and one seventh were men or women in whom infection was attributed to heterosexual intercourse; 76% of all infected cases were male.

The purpose of the present study is to describe the secular trends in incidence of KS in Scotland before and during the AIDS epidemic, to describe geographical variations in incidence in recent years, and to examine the survival of persons with KS.

*Received 17 September 1998*
*Revised 22 October 1998*
*Accepted 28 October 1998*

*Correspondence to:* DH Brewster

### MATERIALS AND METHODS

On the basis of the International Classification of Diseases for Oncology (WHO, 1976) morphology code M-9140/3, incident cases of KS for the years 1976–96 were identified and extracted from the Scottish Cancer Registry (SCR). This registry covers the whole of Scotland (population approximately 5.1 million); data quality is believed to be high, in terms of both accuracy and completeness (Harris et al, 1998). The registry collects a range of patient- and tumour-related information, including demographic and diagnostic details. Every year, mortality data, supplied by the General Register Office (Scotland), are linked to cancer registration records by computerized probability matching (Black et al, 1993).

Mid-year population estimates were derived from the Annual Reports of the Registrar General for Scotland (Registrar General for Scotland, 1977–97). Age-standardized rates were calculated by direct standardization to the World Standard Population (Waterhouse et al, 1976). Within Scotland, the incidence of KS in each health board area of residence was compared to the incidence in Scotland as a whole by indirect standardization to produce standardized incidence ratios (SIR); exact 95% confidence intervals for the SIRs were calculated (Breslow and Day, 1987). Cumulative observed survival was estimated using the Kaplan–Meier method (Kaplan and Meier, 1958).

For comparison, annual numbers of cases of AIDS-defining KS by sex, transmission category and year of diagnosis, covering the period 1986–96 were obtained from the Scottish Centre for Infection and Environmental Health (SCIEH). Scotland's AIDS case-reporting scheme involves consultant clinicians registering patients diagnosed as having AIDS, with SCIEH. An HIV-infected person is defined as having AIDS if he/she has any single opportunistic infection or cancer within an epidemiologically accepted range of such conditions. Information on KS held by SCIEH relates only to cases in which KS was an indicator disease at the time of notification, and does not include subsequent diagnoses of KS.

### RESULTS

In total, 96 cases of KS (82 males) were registered with an incidence date falling within the 21-year period of study (1976–96). Table 1 shows, for all cases (SCR data), and for cases known to be AIDS-defining (SCIEH data), the annual number of cases by sex and year of diagnosis. Of the 45 AIDS-defining cases (SCIEH data), the HIV transmission category was recorded as male homosexual intercourse in 42 cases, and sexual intercourse between men and women in the remaining three cases.

Hereafter, the results relate to all cases of KS (SCR data). Secular trends in age-standardized incidence rates by sex are shown in Figure 1. In males, the incidence rate remained below 0.09 per 100 000 person years at risk during the period 1976–85. The incidence rate then rose steeply, eventually peaking at 0.44 in 1991. Thereafter, incidence declined quite steeply but only to around the rate seen in 1987. In females, the incidence rate remained below 0.06 per 100 000 person years at risk during the whole study period, with no substantial change over time.

Figure 2 shows, for males, changes in age-specific incidence curves for three consecutive 7-year periods of diagnosis (1976–82, 1983–89 and 1990–96). Primarily, this demonstrates a marked shift in age distribution towards younger ages at diagnosis. No such pattern is evident for females (Figure 3).

Within Scotland, for males aged under 50 years during the period 1986–96 the highest incidence was seen in the Lothian Health Board area (which includes the city of Edinburgh) with a standardized incidence ratio (SIR) of 380 (95% confidence intervals 265, 528).

For all patients diagnosed during the period 1976–92, the cumulative observed survival at 5 years was 8.7% for the age group 0–49 years and 49.8% for the age group 50–84 years.

**Table 1** Kaposi's sarcoma in Scotland, 1976–96: annual number of cases by sex and year of diagnosis for all KS (SCR[a] data) and for AIDS-defining KS (SCIEH[b] data)

| Year of diagnosis | Annual number of cases | | | | | |
|---|---|---|---|---|---|---|
| | All KS (SCR data) | | | AIDS-defining KS (SCIEH data)[c] | | |
| | Males | Females | Both sexes | Males | Females | Both sexes |
| 1976 | 2 | – | 2 | – | – | – |
| 1977 | – | 3 | 3 | – | – | – |
| 1978 | 1 | – | 1 | – | – | – |
| 1979 | – | – | – | – | – | – |
| 1980 | – | – | – | – | – | – |
| 1981 | 1 | – | 1 | – | – | – |
| 1982 | – | 1 | 1 | – | – | – |
| 1983 | – | 1 | 1 | – | – | – |
| 1984 | 1 | 1 | 2 | – | – | – |
| 1985 | 1 | 1 | 2 | – | – | – |
| 1986 | 3 | 1 | 4 | 4 | – | 4 |
| 1987 | 5 | – | 5 | 3 | – | 3 |
| 1988 | 4 | – | 4 | 2 | – | 2 |
| 1989 | 2 | – | 2 | 4 | 1 | 5 |
| 1990 | 13 | – | 13 | 7 | – | 7 |
| 1991 | 13 | 1 | 14 | 9 | – | 9 |
| 1992 | 8 | 2 | 10 | 2 | – | 2 |
| 1993 | 10 | – | 10 | 4 | – | 4 |
| 1994 | 6 | 1 | 7 | 5 | 1 | 6 |
| 1995 | 6 | 1 | 7 | 2 | – | 2 |
| 1996 | 6 | 1 | 7 | 1 | – | 1 |
| Total | 82 | 14 | 96 | 43 | 2 | 45 |

[a]Scottish Cancer Registry. [b]Scottish Centre for Infection and Environmental Health. [c]Does not include KS arising in individuals after notification of their AIDS diagnoses to SCIEH.

© Cancer Research Campaign 1999 

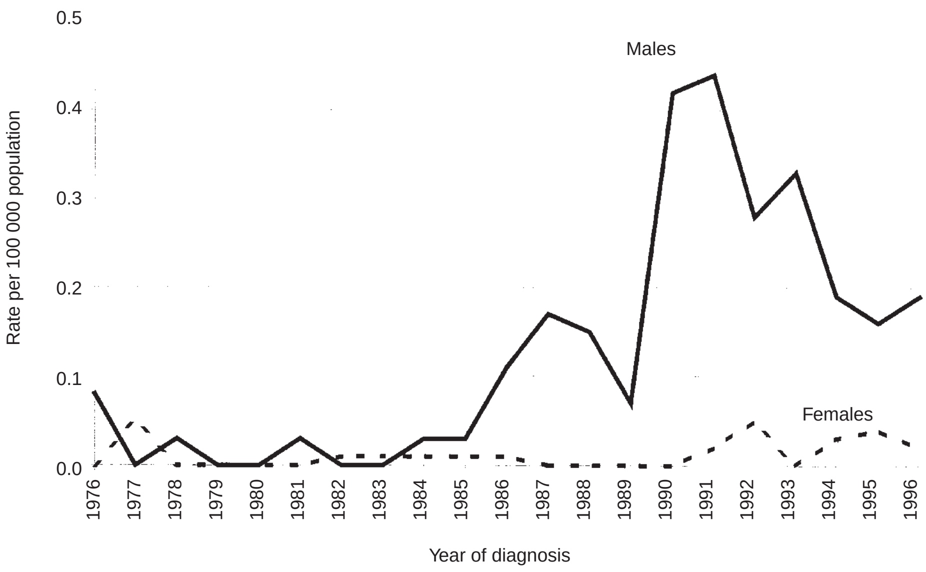

**Figure 1** Kaposi's sarcoma (ICD-O M-9140/3) in Scotland, 1976–96: age-standardized incidence rates (World standard population) by sex and year of diagnosis

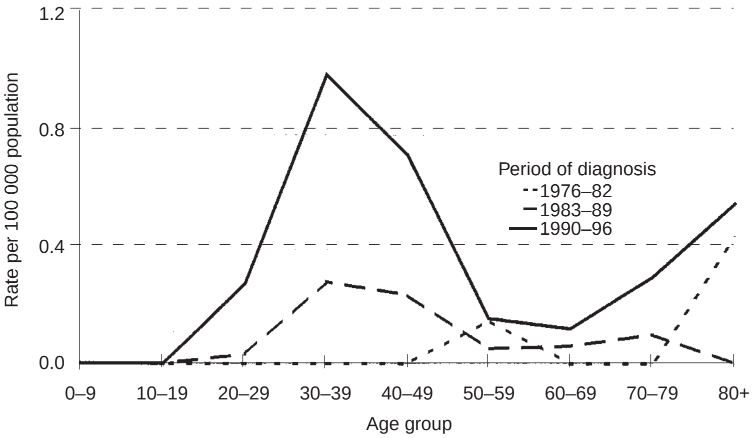

**Figure 2** Kaposi's sarcoma (ICD-O M-9140/3) in Scotland, 1976–96: age-specific incidence rates in males during the periods 1976–82, 1983–89 and 1990–96

*© Cancer Research Campaign 1999*

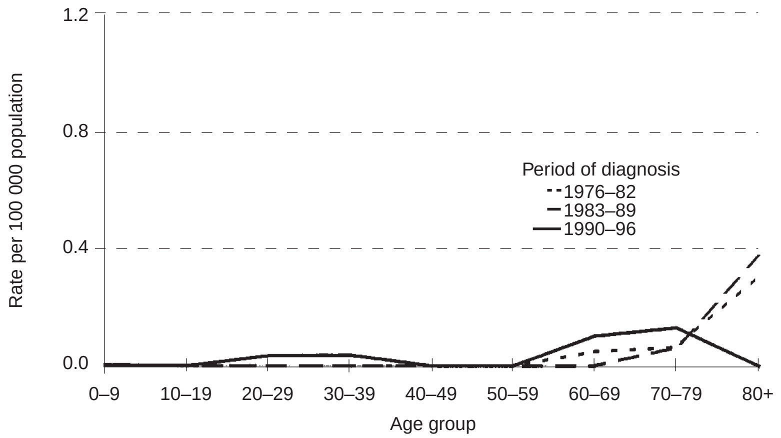

**Figure 3** Kaposi's sarcoma (ICD-O M-9140/3) in Scotland, 1976–96: age-specific incidence rates in females during the periods 1976–82, 1983–89 and 1990–96

### DISCUSSION

As expected, the total number of cases of KS (SCR data) exceeds the number reported to be AIDS-defining (SCIEH data). The apparent excess of AIDS-defining cases in 1989 is likely to reflect different definitions of, or misclassification of, incidence date between the two databases.

Compared to background incidence during the decade 1976–85 (13 cases), the incidence during the subsequent 11 years (83 cases) is higher than can be accounted for by AIDS-defining KS reported to SCIEH (45 cases). However, it is important to remember that SCIEH do not hold data on cases of AIDS-related KS diagnosed after initial notification with a different AIDS indicator disease, such as *Pneumocystis carinii* pneumonia. Other possible explanations for the discrepancy include: under-ascertainment by SCR during the earlier period of observation, leading to under-estimation of the background incidence; registration of some invalid cases by SCR during the more recent period; or a genuine, recent parallel increase in the incidence of classic KS.

The concentration of AIDS-defining KS among individuals who probably acquired HIV through anal sexual intercourse is certainly not a new finding. Historically, AIDS-defining KS has been more common in Britain among homosexual men whose likely source of infection included contacts from the United States or Africa, implying the existence of a sexually transmissible agent or other co-factor distinct from HIV which plays an important part in the aetiology of AIDS-related KS (Beral et al, 1991). This agent is now thought to be Kaposi's-sarcoma-associated Herpesvirus (human Herpesvirus 8), and it has been identified in both HIV-positive and HIV-negative patients (Moore and Chang, 1998).

In a country in which the transmission of HIV has been more common among injecting drug users than male homosexuals, one might not expect a dramatic change in the epidemiology of KS with the advent of AIDS. Nevertheless, the impact of AIDS is clearly discernible in males, both in terms of the increase in age-standardized incidence rates from the mid-1980s onwards, and in terms of the shift in age distribution over time. Despite the impact of AIDS, however, KS remains rare in Scotland, both in the context of other neoplasms, and in international terms. For example, in Scotland for the period 1986–95, 46 944 new cases of lung cancer were registered (Harris et al, 1998) compared with only 76 cases of KS; and, during the period 1988–92, the age-standardized incidence rate of KS in males was 33.5 per 100 000 in 'non-Hispanic whites' from San Francisco compared with 0.3 in Scotland (Parkin et al, 1997).

The fall in incidence among males in Scotland since the early 1990s may reflect the observation that, as a proportion of AIDS-defining illnesses, KS has been reported to have decreased from 1987–89 to 1993–94 in homosexual and bisexual men in all European regions and in the United States (Franceschi et al, 1997). In Scotland, the number of AIDS cases diagnosed rose from 345 to 480 between the periods 1982–91 and 1992–96, but the number in which KS was reported to be an indicator disease fell from 27 (7.8%) to 13 (2.7%) (Allardice et al, 1996), despite a similar number of male homosexuals being diagnosed with AIDS in each period (165 and 172 cases, respectively). This trend may be partly due to the shorter latency period between acquisition of HIV infection and development of KS than is seen between acquisition of HIV and onset of other AIDS-defining illnesses (Hermans et al, 1996). Equally, it might reflect the adoption of safer sexual practices among homosexual men (Dore et al, 1996). The latter hypothesis is supported by falling numbers of new cases of gonococcal infection in men in Scotland from the mid-1980s to a trough in 1994 (Scottish Centre for Infection and Environmental Health, 1998), although recent data suggest that unprotected anal intercourse is still occurring and may be becoming more common again among homosexual men (Young et al, 1997). While the advent of intensive antiretroviral therapies may also now be contributing to the decline in KS incidence (Michaels et al, 1998), it is probably too recent to account for the decline which began in the early 1990s.

The fact that the highest rate of KS in males under 50 years during the period 1986–96 was recorded in Lothian Health Board area is consistent with two observations. First, it reflects the fact

© Cancer Research Campaign 1999

that 45% of all known HIV-infected individuals in Scotland have been reported to be in this area (Goldberg et al, 1998), despite the fact that it contains only approximately 15% of the Scottish population (Registrar General for Scotland, 1997). Second, it is consistent with the fact that Lothian has high rates of rectal gonococcal infection in men compared with Scotland as a whole (Young et al, 1997).

Risk of KS is also increased in organ transplant recipients receiving immunosuppressive therapy (Kinlen, 1982). While the population of organ transplant recipients at risk in Scotland is certainly increasing over time (Junor, 1996; Walker, 1996), the absence of any discernible rise in incidence of KS among females suggests that immunosuppressive therapy following organ transplantation has not played a major part in shaping trends in KS in Scotland.

In contrast to virtually all other neoplasms (Black et al, 1993), survival has been worse in younger than in older age groups in recent years. This reflects the more aggressive nature of AIDS-related KS, as well as the other co-morbidities and competing causes of death which are associated with AIDS. Certainly, median survival for AIDS patients presenting with KS has been consistently reported as between only 19 and 22 months (Allardice et al, 1996).

In conclusion, although the HIV epidemic has had a demonstrable impact on the epidemiology of KS in males in Scotland, KS remains rare in the context of the total burden of cancer in Scotland, and compared with its reported incidence in many other countries.

### ACKNOWLEDGEMENTS

We are grateful to Glenn Codere for extracting data on AIDS-defining KS from the SCIEH database, and to Jim Chalmers for his helpful comments on an earlier draft of the manuscript.

### REFERENCES

Allardice GM, McMenamin J and Goldberg D (1996) AIDS indicator diseases in Scotland (to 31st December 1996). *ANSWER (AIDS News Supplement to the Weekly Report)* **AM-30**: 1–4

Beral V, Bull D, Jaffe H, Evans B, Gill N, Tillett H and Swerdlow AJ (1991) Is risk of Kaposi's sarcoma in AIDS patients in Britain increased if sexual partners came from United States or Africa? *BMJ* **302**: 624–625

Black RJ, Sharp L and Kendrick SW (1993) *Trends in Cancer Survival in Scotland 1968–1990*. Information & Statistics Division: Edinburgh

Breslow NE and Day NE (1987) *Statistical Methods in Cancer Research. Vol. II – The Design and Analysis of Cohort Studies*. IARC Scientific Publication no. 82. International Agency for Research on Cancer: Lyon

Centers for Disease Control (1981) Kaposi's sarcoma and pneumocystis pneumonia among homosexual men. New York City and California. *MMWR* **30**: 305–308

Dore GJ, Li Y, Grulich AE, Hoy JF, Mallal SA, Mijch AM, French MA, Cooper DA and Kaldor JM (1996) Declining incidence and later occurrence of Kaposi's sarcoma among persons with AIDS in Australia: the Australian AIDS cohort. *AIDS* **10**: 1401–1406

Franceschi S, Dal Maso L, Lo Re A, Serraino D and La Vecchia C (1997) Trends of Kaposi's sarcoma at AIDS diagnosis in Europe and the United States, 1987–94. *Br J Cancer* **76**: 114–117

Goldberg D, Davis B, Allardice G, McMenamin J and Codere G (1996) Monitoring the spread of HIV and AIDS in Scotland 1983–1994. *Scot Med J* **41**: 131–138

Goldberg D, Allardice G, McMenamin J, Codere G, Raeside F and Smyth W (1998) Monitoring HIV infection and HIV disease progression in Scotland. *Health Bull* **56**: 645–647

Grulich AE, Beral V and Swerdlow AJ (1992) Kaposi's sarcoma in England and Wales before the AIDS epidemic. *Br J Cancer* **66**: 1135–1137

Harris V, Sandridge AL, Black RJ, Brewster DH and Gould A (1998) *Cancer Registration Statistics Scotland 1986–1995*. ISD Scotland Publications: Edinburgh

Hermans P, Lundgren J, Sommereijns B, Pedersen C, Vella S, Katlama C, Lüthy R, Pinching AJ, Gerstoft J, Pehrson P and Clumeck N for the AIDS in Europe Study Group (1996) Epidemiology of AIDS-related Kaposi's sarcoma in Europe over 10 years. *AIDS* **10**: 911–917

Junor BJR (1996) The need for renal replacement therapy in Scotland. *Health Bull* **54**: 474–485

Kaplan EL and Meier P (1958) Nonparametric estimation from incomplete observations. *J Am Stat Assoc* **53**: 457–481

Kinlen LJ (1982) Immunosuppressive therapy and cancer. *Cancer Surv* **1**: 565–583

Michaels SH, Clark R and Kissinger P (1998) Declining morbidity and mortality among patients with advanced human immunodeficiency virus infection (letter). *New Engl J Med* **339**: 405–406

Moore PS and Chang Y (1998) Kaposi's sarcoma, KS-associated herpesvirus, and the criteria for causality in the age of molecular biology. *Am J Epidemiol* **147**: 217–221

Parkin DM, Whelan SL, Ferlay J, Raymond L and Young J (eds) (1997) *Cancer Incidence in Five Continents*, Vol VII, pp. 846–847. IARC Scientific Publication no. 143. International Agency for Research on Cancer: Lyon

Registrar General for Scotland (1977–97). *Annual Reports 1976–1996*. HMSO: Edinburgh

Scottish Centre for Infection and Environmental Health (1998) Surveillance report: sexually transmitted infections. *Scottish Centre for Infection and Environmental Health Weekly Report* **32**: 106–107

Walker D, Smith IM and Jones IG (1996) Adult heart–lung and lung transplantation in Scotland. *Scot Med J* **41**: 101–103

Waterhouse J, Muir C, Correa P and Powell J (1976) *Cancer Incidence in Five Continents*, Vol. III, pp. 454–459. IARC Scientific Publication no. 15. International Agency for Research on Cancer: Lyon

World Health Organization (1976) *International Classification of Diseases for Oncology*. WHO: Geneva

Young H, Moyes A and Noone A (1997) Gonococcal infections in Scotland 1996. *Scottish Centre for Infection and Environmental Health Weekly Report* **31**: 264–268

*© Cancer Research Campaign 1999*
